# Intraocular nematode with diffuse unilateral subacute neuroretinitis: case report

**DOI:** 10.1186/1471-2415-11-15

**Published:** 2011-06-16

**Authors:** Munira Yusoff, Azma-Azalina Ahmad Alwi, Mariyani Mad Said, Sakinah Zakariah, Zulkifli Abdul Ghani, Embong Zunaina

**Affiliations:** 1 Department of Ophthalmology, Hospital Raja Perempuan Zainab II, 15886 Kota Bharu, Kelantan, Malaysia; 2 Department of Ophthalmology, Universiti Sains Malaysia, 16150 Kubang Kerian, Kelantan, Malaysia

## Abstract

**Background:**

Live intraocular nematode is a rare occurrence. Nematode can migrate actively within the eye, creating visual symptoms and damaging ocular tissue.

**Case presentation:**

A 26-year old man presented with painless reduced vision of the left eye for one week duration. It was associated with floaters. Visual acuity on the left eye was hand movement. Anterior segment examination was normal with normal intra-ocular pressure. Fundus examination showed a live nematode lying subretinally at the macular area with macular oedema and multifocal chorioretinal lesions at peripheral retina. There was no vitritis, vasculitis or any retinal hemorrhage. Systemic examination revealed normal findings and laboratory studies only showed leucocytosis with normal eosinophil count and negative serum toxocara antibody. The diagnosis of introcular nematode with diffuse unilateral subacute neuroretinitis was made. He was treated with oral anti-helminths and a course of oral steroid at a reducing dose. The nematode had died evidenced by its immobility during the treatment and finally disintegrated, leaving macular oedema with mottling appearance and mild hyperpigmentation. Multifocal chorioretinal lesions had also resolved. However despite treatment his visual acuity during follow-up had remained poor.

**Conclusions:**

Cases of intraocular nematode, though not commonly encountered, continue to present the ophthalmologist with the problem of diagnosis and management and hence poorer prognosis to the patient.

## Background

Live intraocular nematode is a rare occurrence and most reports were from India [1-3]. India reported few cases of *Gnathostoma spinigerum *[1,2]. After it gained access to the eyeball, these nematode may localize to the anterior chamber [1], the vitreous [2] or the retina [3,4]. Nematode can migrate actively within the eye, creating visual symptoms and damaging ocular tissue. Inflammation and degeneration of the posterior retina related to subretinal migration of nematode is described as diffuse unilateral subacute neuroretinitis [5] and usually results in severe loss of vision [5,6].

## Case Presentation

A 26-year old man from the outskirt of Kota Bharu in Kelantan presented with sudden onset of reduced vision of the left eye for one week duration. Initially it was a central field loss which had then progressively involved the whole visual field. It was associated with floaters but was painless with no eye redness, itchiness or discharge. He had four cats at home which he had a very close contact with. He denied any trauma to the eye or any eye injury and he had no past ocular history or medical illness.

**Figure 1:**
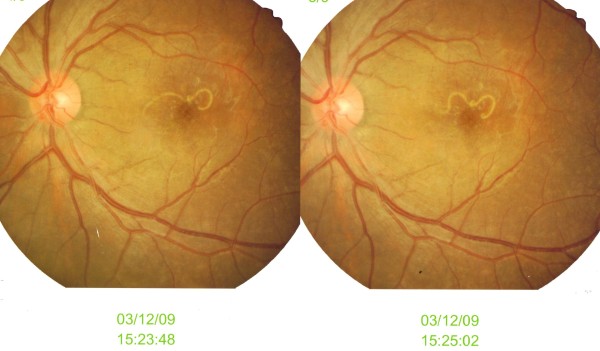
**Left fundus shows migrating nematode at macular area**.

His visual acuity was hand movement on the left eye with presence of relative afferent pupillary defect and 6/6 on the right eye. Left eye examination showed no inflammation in the anterior segment or the vitreous cavity. Funduscopic examination disclosed a white live nematode, approximately two disc diameters in length, moving slowly in the macula at the subretinal space (Figure 1). The body of the nematode was roughly tapered at one end and slightly rounded at the other end. There was presence of macular oedema and multifocal chorioretinal lesions at the peripheral retina. There was no evidence of worm track found clinically, no vasculitis or any retinal hemorrhage. However, fundus fluorescein angiography and visual evoked potential was not done in this patient. The right eye findings were normal. Systemic examination showed no significant finding with no jaundice or hepatosplenomegaly.

The blood investigations revealed white blood count of 10.4 × 10^9^/L (high normal) with normal eosinophil count (0.12 × 10^9^/L), hemoglobin of 17.0 g/L, red blood cell count of 6.38 × 10^12^/L (increased) and normal platelet count. The erythrocyte sedimentation rates (ESR), Mantoux test, liver function test, serum urea and electrolytes as well as his chest x-ray were also normal. Blood for serum toxocara antibody was negative.

**Figure 2:**
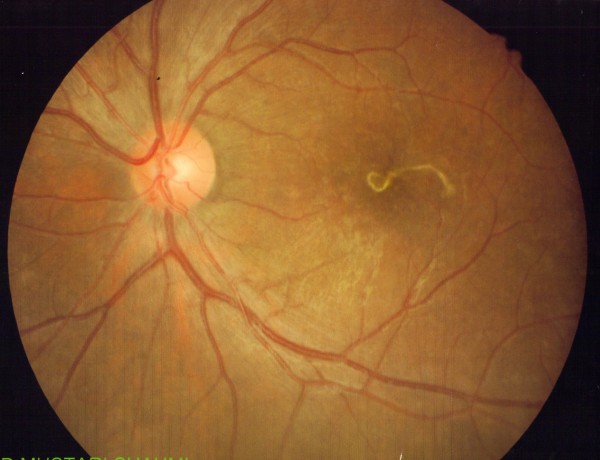
**The nematode was not showing any movement anymore on day 2 of antihelminth**.

**Figure 3:**
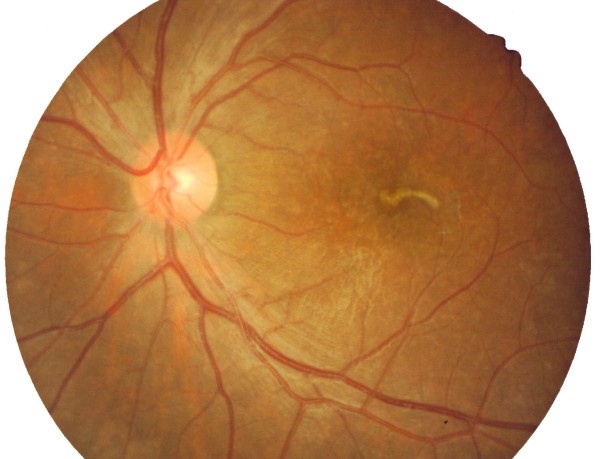
**The nematode appeared less well-defined, with some spots of mild hyperpigmented retina surrounding it after completion 5 days of antihelminth**.

Clinically he was diagnosed to have intraocular nematode with diffuse unilateral subacute neuroretinitis. He was treated with oral albendazole 400 mg 12 hourly for five days based on recommendation by the infectious disease specialist. Two days after the treatment, the nematode was immobile, but its morphology and the rest of the retina remained the same (Figure 2). After completed five days of antihelminth, the nematode had disintegrated, leaving a mottled appearance of the macula with mild hyperpigmentation and resolving chorioretinal lesions (Figure 3). At the same time, oral prednisolone 30 mg perday for a week was also started in order to reduce the inflammation that might caused by toxins liberated from the dead nematode. Then, the dose of prednisolone was tapered down by 5 mg per week. The patient was discharged after one week with oral prednisolone of 25 mg/day. Upon discharge, his left eye visual acuity remains the same with no vitritis and resolved chorioretinal lesions.

## Conclusions

Humans commonly acquire the infection by ingesting contaminated meat or water containing the third-stage larvae. In this patient, he might have infected the organism from his close contact with his cats at home.

This larva will continue its life cycle in human body which include the eye and incite ocular damage by a combination of mechanical, immunologic, and allergic reactions. Local inflammatory changes may be related to toxic effects or immunologic stimulation from excretory products of the larva or from release of unknown soluble tissue toxins. Inflammation and degeneration of the posterior retina related to subretinal migration of nematode is described as diffuse unilateral subacute neuroretinitis [5]. The ocular findings include visual loss, vitreous cells, optic disc inflammation and leakage, and transient recurrent crops of gray-white outer retinal lesions. Later in the course of the disease, slowly progressive retinal pigment epithelium changes and optic atrophy may be observed, as well as narrowing of the retinal vessels [5]. In this patient, the nematode was found migrating in the subretinal space and might cause damage to the outer retina and retinal pigment epithelium. Loss of vision with the presence of relative afferent pupillary defect in the affected eye might be due to death of ganglion cells and neural fibers that lead to damage to the optic nerve.

Diffuse unilateral subacute neuroretinitis has been reported initially in America [7], and later in many other countries, including China [8], Brazil [6], and India [9]. This condition occurs more frequently in males than in females and most frequently in the second and third decade [6].

Symptom relief depends on identification and removal of the nematode. However this is often difficult due to the migratory nature of the live nematode. Various types of management for intraocular nematode have been reported. Previously, the conventional treatment was surgical removal [1]. There are intravitreal or preretinal or subretinal nematode that were retrieved successfully by pars plana vitrectomy in several reported cases [2,4]. This is true for instance if the nematode is lying at the macula because other modes of treatment like photocoagulation may damage the macula. In some cases the nematode may elude capture, creating serious ocular complications. Laser photocoagulation showed successful result and can be done when the nematode moves away from the macula [3].

Antihelminthic treatment is being used more frequently [9]. High dose oral albendazole seems to be safe and beneficial for patients with active diffuse unilateral subacute neuroretinitis in the early or late clinical stage. In this patient, he was treated with albendazole and prednisolone was added to avoid inflammatory response [6,9]. Photocoagulation or removal of nematode through vitrectomy was not done because it would jeopardize the macula and worsen his vision.

Cases of intraocular nematode, though not commonly encountered, continue to present the ophthalmologist with the problem of diagnosis and management and hence poorer prognosis to the patient.

## Competing interests

The authors declare that they have no competing interests.

## Authors' contributions

MY examined, evaluated the patient and wrote the manuscript. AAAA, MMS, SZ and ZAG examined and evaluated the patient. ZE edited the manuscript. All authors read and approved the final manuscript.

## Pre-publication history

The pre-publication history for this paper can be accessed here:

http://www.biomedcentral.com/1471-2415/11/15/prepub

## References

[B1] Tiwari S Chayani N Rautaraya B Intraocular Gnathostoma spinigerum: a case report Cases J 2009 2 9370 10.1186/1757-1626-2-9370 20062613 PMC2804013

[B2] Basak SK Sinha TK Bhattacharya D Hazra TK Parikh S Intravitreal live gnathostoma spinigerum Indian J of Ophthalmol 2004 52 57 58 15132381

[B3] Ittyerah TP Nematode in the retina Indian J of Ophthalmol 1990 38 4 178 179 2086470

[B4] Yamamoto S Hayashi M Takeuchi S Surgically removed submacular nematode Br J of Ophthalmol 1999 83 9 1088 10.1136/bjo.83.9.1088 PMC1723183 10636682

[B5] Gass JDM Gilbert WR Guerry RK Scelfo R Diffuse unilateral subacute neuroretinitis Ophthalmology 1978 85 521 545 673332 10.1016/s0161-6420(78)35645-1

[B6] Garcia CA Gomes AHB Garcia Filho CA Vianna RNG Early stage diffuse unilateral subacute neuroretinitis: improvement of vision after photocoagulation of the worm Eye 2004 18 624 627 10.1038/sj.eye.6700742 14716322

[B7] Cunha de Souza E Lustosa da Cunha S Gass JDM Diffuse unilateral subacute neuroretinitis in South America Arch Ophthalmol 1992 110 1261 1263 1520112 10.1001/archopht.1992.01080210079029

[B8] Cai J Wei R Zhu L Cao M Yu S Diffuse unilateral subacute neuroretinitis in China Arch Ophthalmol 2000 118 721 722 10815171

[B9] Myint K Sahay R Mon S Saravanan VR Narendran V Dhillon B Worm in the eye: the rationale for treatment of DUSN in south India Br J Ophthalmol 2006 90 1125 1127 10.1136/bjo.2006.094490 16707523 PMC1857373

